# Shifting Perceptual Weights in L2 Vowel Identification after Training

**DOI:** 10.1371/journal.pone.0162876

**Published:** 2016-09-20

**Authors:** Wei Hu, Lin Mi, Zhen Yang, Sha Tao, Mingshuang Li, Wenjing Wang, Qi Dong, Chang Liu

**Affiliations:** 1 State Key Laboratory of Cognitive Neuroscience and Learning & IDG/McGovern Institute for Brain Research, Beijing Normal University, Beijing, China; 2 National Innovation Center for Assessment of Basic Education Quality, Beijing Normal University, Beijing, China; 3 Department of Communication Sciences and Disorders, University of Texas at Austin, Austin, Texas, United States of America; Sun Yat-Sen University, CHINA

## Abstract

Difficulties with second-language vowel perception may be related to the significant challenges in using acoustic-phonetic cues. This study investigated the effects of perception training with duration-equalized vowels on native Chinese listeners’ English vowel perception and their use of acoustic-phonetic cues. Seventeen native Chinese listeners were perceptually trained with duration-equalized English vowels, and another 17 native Chinese listeners watched English videos as a control group. Both groups were tested with English vowel identification and vowel formant discrimination before training, immediately after training, and three months later. The results showed that the training effect was greater for the vowel training group than for the control group, while both groups improved their English vowel identification and vowel formant discrimination after training. Moreover, duration-equalized vowel perception training significantly reduced listeners’ reliance on duration cues and improved their use of spectral cues in identifying English vowels, but video-watching did not help. The results suggest that duration-equalized English vowel perception training may improve non-native listeners’ English vowel perception by changing their perceptual weights of acoustic-phonetic cues.

## Introduction

It is well known that phonemic perception in a second language is quite challenging [1–4]. For a native Chinese speaker learning English, vowel perception is more difficult than consonant perception [5–7]. In particular, English back vowels are more difficult to perceive than front vowels for non-native English listeners [8–10]. For example, native Chinese listeners correctly identified English back vowels much less frequently than other English vowels [9, 10].

Phonemic perception in a second language may be improved with perceptual training for non-native listeners. Most phonemic perception training studies have focused on consonant identification by using certain pairs of consonants that were easily confused by non-native listeners. With ten-hour training on identifying and distinguishing the English /r/-/l/ pair in synthetic speech (e.g., rock-lock), native Japanese listeners’ perception of English /r/-/l/ significantly improved [11]. The training effect also extended to other untrained synthetic phonetic contexts, such as rake-take. Logan et al. [12] confirmed these findings with natural speech materials and reported that the training effect transferred to new words and new talkers. Moreover, perceptual training that incorporated a wide variety of words and talkers had significantly improved native Japanese listeners’ perception of English consonants /r/-/l/, and the training effect was retained for three months post-training [13]. These studies suggest that a variety of training stimuli may lead to significant improvement in second-language learners’ consonant perception.

In addition to the investigation of consonant perception training, several phonetic training studies were conducted on non-native listeners’ vowel perception. Nishi and Kewley-Port [14] trained Japanese learners of English for more than 13 hours with two paradigms: one with nine American English (AE) monophthongs /i, I, ε, æ, a, ʌ, ɔ, u, U/ in nonsense words, and the other with only the three most difficult vowels /a, ʌ, u/, which were correctly identified much less frequently (41%) than the other vowels (59%) at pre-test in nonsense words. The results showed that the learners trained with nine vowels improved their identification for all vowels and increased performance retention after three months, whereas the learners trained with three vowels only improved their perception of the three trained vowels. Another study trained Portuguese learners of English on English vowel perception for five hours, which involved six target vowels (/i/-/I/, /æ/-/e/, /u/-/U/) in real words [8]. The results showed that the training effect of the two front vowel pairs, but not the back vowel pair, transferred to new words and new talkers, indicating that it might be more difficult to generalize the training benefits to new stimuli and new talkers for the back vowel contrasts. These results revealed that vowel perceptual training improved the vowel identification of non-native listeners, but the training effect may be dependent on vowel category. In the present study, five English back vowels /ʌ, U, u, ɔ, ɑ/ were included in a six-hour training to examine whether such training could improve native Chinese listeners’ English back vowel identification and whether the training effect could be extended to those untrained vowels.

Non-native learners’ difficulties with second-language vowel perception may stem from their challenges with using acoustic-phonetic cues. Non-native English listeners, such as native Arabic, Japanese and Spanish listeners, rely mainly on duration cues to identify English vowels [15, 16]. Recent studies reported that native Chinese listeners also relied more on vowel duration for English vowel perception compared with native English listeners [5, 17]. Moreover, studies showed that training with stimuli of equalized duration improved vowel perception [18, 19]. Ylinen et al. [18] compared the identification of the English vowels /i/ and /I/ by native Finnish listeners before and after training. Stimuli with equalized duration were used in the training. The results showed that before the training the identification for /i/ of equalized duration was significantly lower than that of natural duration. However, after the training, the identification for /i/ of equalized duration had significantly improved compared with the pre-training performance, although it was still lower than that of natural duration. Giannakopoulou et al. [19] replicated the results with native Greek speakers learning English as a second language. These results were consistent with Francis’s attention-to-dimension model [20]; that is, the change of attention in a cue-dependent manner essentially affects perceptual learning. In other words, withdrawing attention from the duration cues for non-native learners may shift the perceptual weight to vowel spectrum and finally improve their phonemic perception in second-language learning.

In addition to vowel duration, vowel formant frequency may play a critical role in vowel identification and categorization [21, 22]. Kewley-Port et al. [23] revealed a moderately negative correlation between English vowel formant discrimination and vowel identification across four language groups such as native-American English, Swedish, Danish, and Japanese listeners. In other words, the lower the sensitivity to vowel formant frequency change, the lower the listener’s vowel identification scores. These results suggested that compared with native listeners, non-native listeners may be less sensitive to the formant frequency change of non-native vowels, possibly resulting in a less efficient use of formant frequency cues and their poor identification of non-native vowels. Moreover, native Chinese listeners had significantly higher vowel formant discrimination thresholds (i.e., lower sensitivity to formant frequency change) compared with their native English counterparts [24]. Such lower sensitivity to formant frequency change may be associated with greater difficulty using formant frequency cues to identify vowels. Phonetic training may help second-language learners enhance their sensitivity in detecting vowel formant frequency change and thus lead to better vowel identification. In the present study, the relationship between vowel identification and vowel formant discrimination was examined. Another goal of this study was to investigate whether vowel perception training could improve Chinese listeners’ sensitivity to formant frequency change and improve their ability to use formant frequencies to perceive English vowels.

The objectives of the present study were to explore (1) whether perceptual training of English back vowels with equalized duration could reduce native Chinese listeners’ dependency on vowel duration and improve their English vowel formant discrimination compared with a control group with general language input (i.e., video watching); and (2) whether English vowel perceptual training without duration cues may reduce reliance on duration cues and improve the use of spectral cues for non-native learners. We recruited two groups of native Chinese listeners with comparable English learning experience and proficiency: one was the training group with English back vowel perception training; the other was the control group assigned to watch an English video. Vowel identification and vowel formant discrimination were measured before, immediately after, and three months after the training.

## Materials and Methods

### Participants

Thirty-four students (20 females and 14 males, mean age 22.9) from Beijing Normal University participated in the experiment. All listeners were native Mandarin Chinese listeners and started learning English at school when they were 11 or 12 years old. All listeners passed the College English Test Band 4 (CET-4) in China. The CET-4 is required for Chinese undergraduate students to receive a Bachelor’s degree in most universities in China. No listeners reported a residence history in English-speaking countries. All listeners had normal hearing with pure-tone thresholds ≤ 15 dB HL at octave intervals between 250 and 8000 Hz [25]. All participants signed informed consent forms and were paid for their participation. The procedures were approved by the Research Ethics Board of Beijing Normal University.

**Fig 1:**
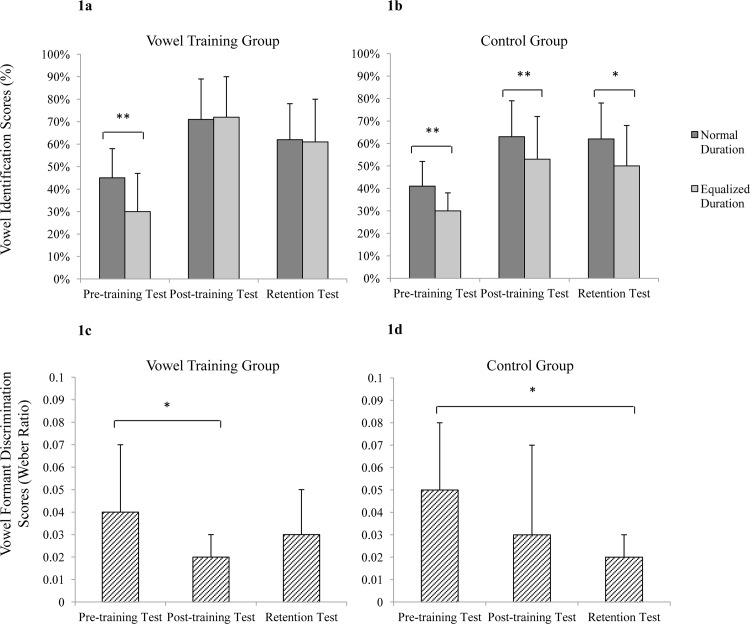
**Vowel identification scores in correct-response percentage of normal duration, equalized duration of trained vowels in the pre-training test, post-training test, and retention test of the vowel training group (Fig 1a: Upper left) and of the control group (Fig 1b: Upper right). Vowel formant discrimination scores of /ʌ/ in the pre-training test, post-training test, and retention test of the vowel training group (Fig 1c: Bottom left) and of the control group (Fig 1d: Bottom right).** Error bars represent the standard deviations of the means. * Symbol represents significant differences: *p* < 0.05; and ** represents *p* < 0.01.

Participants were randomly assigned to one of two groups (i.e., the vowel-training group or the control group) with matched age (23.47 ± 1.55 versus 23.06 ± 2.08, *df* = 16, *t* = 0.65, *p* = 0.519), age of English acquisition (11.25 ± 1.88 versus 11.12 ± 1.93, *df* = 16, *t* = 0.20, *p* = 0.844), CET-4 score (514.31 ± 43.83 versus 517 ± 39.79, *df* = 16, *t* = -0.19, *p* = 0.853), and pre-training test performance (see Fig 1).

### Stimuli and apparatus

Twelve English monophthongs /æ, ε, e, i, I, з, ʌ, U, u, o, ɔ, ɑ/ served as the speech stimuli in the vowel identification test. All monophthongs were originally recorded in the syllable context of /hVd/ (e.g., had, hawed, hayed, head, heed, heard, hid, hod, hoed, hood, hud, and who’d) produced by a young female native speaker of American English from the state of Texas, USA. Each stimulus had two versions: one with normal vowel duration and the other with equalized vowel duration. The vowels with normal duration were obtained by eliminating the initial /h/ and ending /d/ with the duration range from 186 to 294 ms; the vowels with equalized durations were edited by removing the onset and offset formant transitions of the syllable with the central vowel nucleus, thereby shortening the vowel duration to 170 ms. The vowel stimuli had 10-ms rise-fall ramps. The sound pressure level of all vowels was 70 dB and was calibrated in an AEC201-AIEC 60318–1 ear simulator by a Larson-Davis sound-level meter (Model 2800) with a linear weighting band.

A recent study found a moderately significant correlation between the thresholds of vowel formant discrimination of /ʌ/ and the identification performance of English vowels in equalized duration condition for native Chinese listeners [9]. The isolated American English vowel /ʌ/ with an equalized duration of 170 ms, which was used for the vowel identification experiment, served as the speech stimulus for vowel formant discrimination. Furthermore, Liu et al. [24] reported that the most significant difference in vowel formant discrimination between native Mandarin Chinese and native English listeners was at F2, but not at F1. Thus, only the F2 frequency discrimination of /ʌ/ was tested in this study.

Speech stimuli were presented via SONY MDR-7506 headphones to the listeners, who were seated in a quiet test room. In order to compare the results between the current study and previous studies [9, 24, 26, 27], stimuli were played to the right ear of the listeners in the present study. Stimulus presentation was controlled by a Tucker-Davis Technologies mobile processor (RM1) with a compatible sampling frequency at 12,207 Hz. Sykofizx^®^ software was used to implement all test and training procedures.

### Procedure

First, all participants completed English vowel identification and vowel formant discrimination tasks and English-learning experience questionnaires. Participants were then randomly assigned to either of the two groups. Second, participants in the experimental group received training one hour per day for six consecutive days, while participants in the control group watched videos for one hour per day for six consecutive days. Third, after completing the training or video-watching sessions, all participants completed English vowel identification with a new talker and vowel formant discrimination tasks. Fourth, three months after the training participants returned for a retention test.

### Pre- and post-training tests

Vowel identification task: Twelve response alternatives were presented on a computer screen as a text box labeled with the /hVd/ context (e.g., had, hawed, hayed, head, heed, heard, hid, hod, hoed, hood, hud, and who’d) corresponding with each vowel. After each vowel presentation, participants were asked to identify the vowel within 10 seconds by clicking on the text box corresponding with their response choice.

Under each test condition (normal or equalized duration), vowel identification was measured in one block of 240 trials, 20 for each vowel in a random order for each listener. The sequence of the two conditions (normal and equalized duration) was randomized across listeners. To familiarize participants with the procedure before data collection, participants practiced with a 15-min session of vowel identification using vowels produced by two native-English male talkers. Vowels were presented in isolation for the test sessions.

After the post-training test, listeners were tested again to assess the degree to which the training effect generalized to stimuli produced by a new male native-English talker from the state of Texas, USA. The test stimuli and the identification task were identical to the procedures used in the pre-training test, post-training test, and retention test. Vowel identification was measured in one block of 120 trials, ten for each vowel in a random order for each listener. The test was repeated again three months after the training.

Vowel formant discrimination task: Thresholds for formant discrimination were measured for the F2 of isolated English vowel /ʌ/ with an equalized duration of 170 ms using a three-interval, two-alternative forced-choice procedure, with a two-down, one-up tracking algorithm, estimating 70.7% correct responses [28]. Specifically, for each test trial there were three intervals with the standard stimulus presented in the first interval, followed by a standard stimulus and a formant-shifted stimulus randomly ordered in the second and third intervals. The inter-stimulus interval (ISI) was 400 ms, consistent with previous studies of formant discrimination [29, 30]. Listener’s task was to indicate which of the two test intervals contained the stimulus that sounded different from the standard one by pressing the appropriate button. Listeners had 10 s to respond after the presentation of three intervals for each trial. Feedback was provided after each response was collected. The next trial began automatically 1 s afterwards. For each block, the formant shift, starting at 20% of the formant frequency, was adjusted in 2.5% steps for the first three reversals and in 0.5% steps thereafter. The threshold was computed as the average formant shift value corresponding with the remaining even number of reversal points. The threshold for each condition was the average of two 60-trial blocks, unless the formant thresholds for the two blocks differed by more than 1% of the target formant frequency (e.g., two 0.5% steps), in which case a third block was presented. Thus, there were 4–6 blocks for each listener and the first four experimental blocks (e.g., two blocks of each vowel) were interleaved, followed by the third blocks if needed.

### Training session

The total training time for vowel perception was six hours, which was divided into six one-hour sessions in six consecutive days. For the vowel training group, participants were asked to complete vowel identification in which duration-equalized vowels were used. Five English back vowels /ʌ, U, u, ɔ, ɑ/ produced by six native English talkers (three males and three females from the state of Texas, USA) were presented in isolation for the training session; the other seven English monophthongs /æ, ε, e, i, I, з, o/ served as the untrained stimuli in the test sessions. A variety of talkers were used in the training sessions, because previous studies found that the first and second formant frequencies varied widely among speakers [22] and the perceptual training with a large number of talkers significantly improved non-native listeners’ phonetic perception [13]. All talkers in the training session were between the ages of 20–28 and were different from the talkers in the test sessions. Each training session consisted of 18 blocks; for each block the five back vowels were presented ten times each in random order. Feedback was provided for each trial during the training sessions, but not during the test sessions.

For the control group, participants were asked to watch the television sitcom *Friends* without subtitles for one hour per day. Next, they were required to answer multiple-choice questions (e.g., everybody celebrated in ______’s engagement night together. *a*. *Ross; b*. *Rachel; c*. *Phoebe; d*. *Monica*.) that were relevant to the video.

## Results

### Training effect on vowel identification

To minimize the floor and/or ceiling effect on the statistical results, the percent-correct identification scores were converted to rationalized arcsine units (RAUs) [31]. Fig 1 reports the descriptive data for vowel identification and vowel formant discrimination in the pre-training test, post-training test, and retention test of both the vowel training group and the control group.

#### Improvement and retention in vowel identification

For trained vowels: A three-way (within-subjects factors: vowel duration × test time; between-subjects factor: group) repeated-measures ANOVA was performed. Bonferroni correction was used for multiple comparisons of the ANOVA to control Type I error. The results showed significant main effects of test time (*F*_2, 52_ = 39.81, *p* < 0.001, η_p_^2^ = 0.605) and vowel duration (*F*_1, 26_ = 19.70, *p* < 0.001, η_p_^2^ = 0.431), and significant interaction effects between the three factors (*F*_2, 52_ = 3.24, *p* = 0.047, η_p_^2^ = 0.111) and between test time and vowel duration (*F*_2, 52_ = 3.22, *p* = 0.048, η_p_^2^ = 0.110). However, no significant main effect of group (*p* = 0.284) and no significant interaction effects between test time and group (*p* = 0.398) or between vowel duration and group was found (*p* = 0.198).

For untrained vowels: A similar ANOVA was performed on untrained vowels. The results showed a significant main effect of test time (*F*_2, 52_ = 17.96, *p* < 0.001, η_p_^2^ = 0.409), while no significant effects of vowel duration, group, and multi-factor interactions were observed (three-factor interaction: *p* = 0.090; interaction between duration and group: *p* = 0.672; interaction between time and group: *p* = 0.073; interaction between duration and time: *p* = 0.399).

#### Training effect on the vowel identification of a new talker

For trained vowels: A two-factor (within-subjects factor: test time; between-subjects factor: group) ANOVA was conducted with the vowel identification score of equalized duration condition only as the dependent variable. The main effect of group was significant (*F*_1, 26_ = 5.83, *p* = 0.023, η_p_^2^ = 0.183). In particular, in the post-training test, listeners in the vowel training group scored approximately 22% higher than those listeners in the control group in identifying vowels spoken by a new talker. After three months, the vowel training group still showed 13% better performance than the control group. The main effect of test time was significant (*F*_1, 26_ = 6.64, *p* = 0.016, η_p_^2^ = 0.203), but the interaction effect between test time and group was not significant (*p* = 0.110).

For untrained vowels: A similar ANOVA analysis was performed on untrained vowels. No significant main effects were found (group: *p =* 0.070; test time: *p* = 0.331), and no significant interaction effect between test time and group was observed (*p* = 0.980).

### Training effect on vowel formant discrimination

The thresholds of formant discrimination were represented as the Weber ratio (ΔF/F). The Weber ratio was used to facilitate statistical comparisons among different vowels in previous studies [24, 26], although threshold was examined for only one vowel formant frequency in this study. A two-way (test time ×group) ANOVA was performed. The results showed a significant main effect of test time (*F*_2, 52_ = 6.238, *p* = 0.004, η_p_^2^ = 0.193), while there was no significant main effect of group (*p* = 0.929). Moreover, no significant two-factor interaction effect was found (*p* = 0.773).

### Training effect on perceptual weights

#### Duration effect change in vowel identification

The duration effect was computed as the difference between the vowel identification performance of normal duration and that of equalized duration. Cohen’ d was further calculated to examine the effect size. The formula for calculating the effect size of the duration effect is: d = (M_1_—M_2_) / SD_pooled_, where M_1_ and M_2_ are means of vowel identification scores of normal and equalized duration vowels from the same group of participants, respectively, and SD_pooled_ is the pooled standard deviation of the two conditions [32].

For trained vowels: From a three-way (within-subjects factors: vowel duration × test time; between-subjects factor: group) ANOVA that was performed in 3.1.1 for trained vowels, a significant three-factor interaction effect was obtained (*F*_2,52_ = 3.24, *p* = 0.047, η_p_^2^ = 0.111).

**Fig 2:**
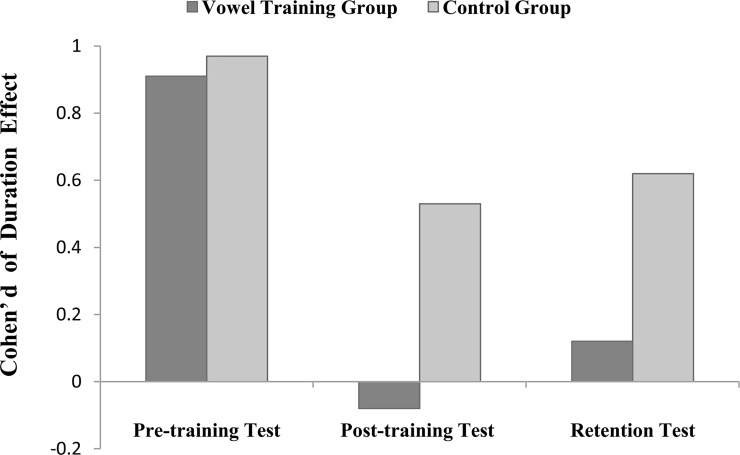
Cohen's d of the duration effect for both the vowel training group and the control group collapsed over the test time (pre-training, post-training, and retention tests).

For the vowel training group, a subsequent simple-effect analysis revealed a significant duration effect (more than 15%, *p* = 0.006, Cohen’s d = 0.91) before the training, but no significant duration effect (less than -1%, *p* = 0.457, Cohen’s d = -0.08) after the vowel training, indicating that the listeners in the vowel training group reduced their reliance on vowel duration for the identification task after training. In addition, a non-significant duration effect (less than 2%, *p* = 0.538, Cohen’s d = 0.12) in the retention test indicated that this reduced duration effect was maintained for three months after training. In contrast, listeners in the control group consistently showed significant duration effects in the pre-training test (11%, *p* = 0.005, Cohen’s d = 0.97), the post-training test (10%, *p* = 0.008, Cohen’s d = 0.53), and the retention test (12%, *p* = 0.021, Cohen’s d = 0.62), suggesting that a general English input such as video-watching did not change Chinese listeners’ heavy reliance on duration for vowel perception (see Fig 2).

For untrained vowels: From a three-way (within-subjects factors: vowel duration × test time; between-subjects factor: group), repeated-measures ANOVA that was performed in 3.1.1 for untrained vowels, neither the main effect of vowel duration nor the interactions between vowel duration and group was significant (*ps* > 0.05).

#### Training effect on the relationship between vowel identification and vowel formant discrimination

**Fig 3:**
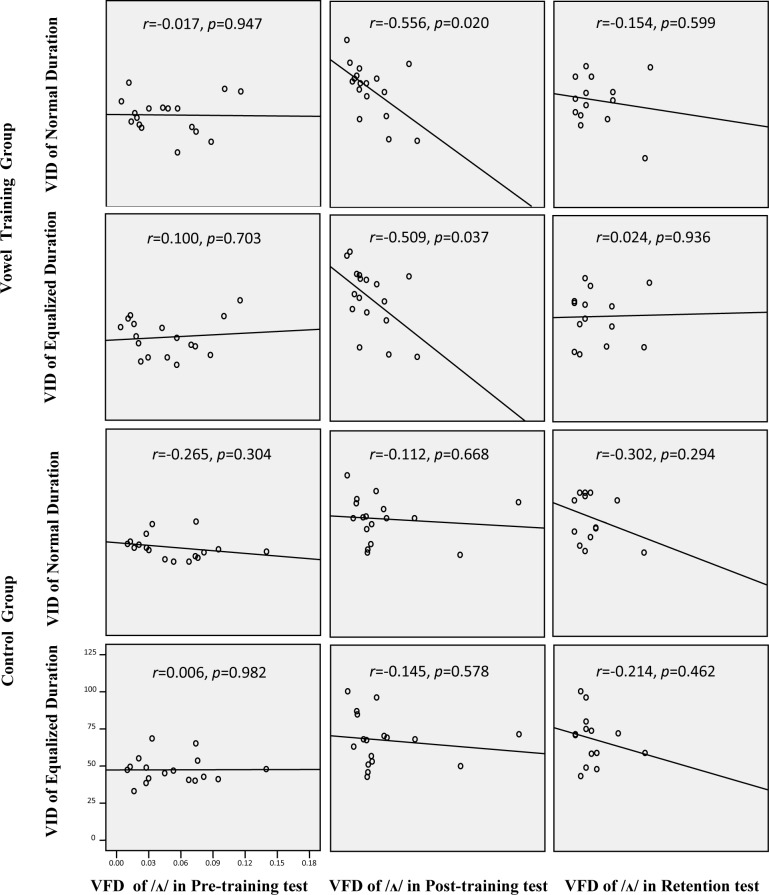
The correlation between the scores of the vowel identification (VID) of normal or equalized duration and the thresholds of vowel formant discrimination (VFD) of /ʌ/ in the pre-training, post-training, and retention tests of both the vowel training group and the control group.

A series of correlation analyses were conducted between the formant discrimination thresholds of /ʌ/ and the performance of vowel identification in normal or equalized duration condition for the pre-training, post-training, and retention tests (see Fig 3). The data related to vowel identification from all 12 vowels were pooled in the correlation analysis.

For the vowel training group, the vowel formant discrimination of /ʌ/ did not significantly correlate with the performance of vowel identification in both normal (*r* = -0.017, *p* = 0.947) and equalized duration conditions (*r* = -0.100, *p* = 0.703) in the pre-training test. Remarkably, in the post-training test the correlations became significant in both the normal (*r* = -0.556, *p* = 0.020) and equalized duration conditions (*r* = -0.509, *p* = 0.037). The significant negative correlations meant that the lower the threshold of formant frequency change, the higher the vowel identification performance. Such changes in the post-training test suggest that the perceptual weight had shifted toward the formant cues for vowel identification based on vowel training; however, the significant correlation was not in the retention test in either the normal (*r* = -0.154, *p* = 0.599) or equalized duration conditions (*r* = 0.024, *p* = 0.936).

For the control group, no significant correlation was found in both the normal and equalized duration conditions in the pre-training test, post-training test, and retention tests (all *ps* > 0.05). This finding suggests that there was no shift of perceptual weight in the formant and duration cues for vowel perception with the control group.

## Discussion

In this study native Chinese listeners were assigned to two separate groups: the vowel perception training group (i.e., identifying English vowels) and the control group (i.e., video-watching group). The results showed significant improvements for both groups in English vowel identification and vowel formant discrimination, with the improvement retained after three months. Meanwhile, the training effect was transferred to untrained vowels and to vowels produced by a new talker. In addition, the vowel perception training not only successfully reduced English learners’ reliance on vowel duration in English vowel perception, it also improved learners’ use of spectral cues in vowel perception (i.e., greater correlations between vowel identification and vowel formant discrimination). In contrast, a general English speech input such as video-watching did not reduce English learners’ reliance on duration in vowel perception (i.e., no significant relationship between vowel identification and vowel formant discrimination was observed before and after training).

## Training effect on vowel identification

Although both the vowel training group and the control group improved their English vowel identification, the vowel training group (e.g., 33%) showed greater improvement than the control group (e.g., 23%). Moreover, the vowel training group showed better performance than the control group in identifying English vowels from a new talker right after (e.g., 22%) and three months after training (e.g., 13%).

Furthermore, compared with previous vowel training studies, which found no perceptual improvement on untrained vowels [8, 14], this study showed a significant training effect to untrained vowels for both the vowel training group and the control group, probably because a greater number of vowels were trained in this study. Specifically, Nishi and Kewley-Port [14] trained Japanese learners to identify three English central/back vowels /ɑ, ʌ, u/, and Rato [8] trained Portuguese learners to identify English back vowel pair /u/-/U/; in contrast, the present study trained native Chinese listeners to identify five English back vowels, primarily because the back vowels were identified less accurately than front and central vowels [9]. These results suggest that to improve English vowel perception for non-native listeners, a greater number of vowels, if not the entire inventory, are needed in perception training.

## Training effect on vowel formant discrimination

This study revealed that short-term training of vowel identification significantly improved non-native listeners’ vowel formant discrimination. Native Chinese listeners improved their English vowel formant discrimination immediately after vowel training with equalized duration; however, this improvement was not observed in the retention test, suggesting that more training may be required. On the other hand, vowel formant discrimination also improved for the control group, indicating that general English speech input may enhance formant discrimination regardless of the low-occurrence frequency of the vowel /ʌ/ in general American English [33]. However, it should be noted that the relationship between vowel identification and vowel formant discrimination changed from non-significant in the pre-training to significant in the post-training test only for the vowel training group and remained non-significant for the control group. These results suggest that although both vowel training and video-watching can improve sensitivity to vowel formant frequency change, only the vowel training group could have the better formant discrimination lead to higher vowel identification.

## Training effect on perceptual weights

Although both training groups showed significant improvements in vowel perception with training, the mechanisms for the improvement may differ between the two groups. The advantage of vowel training with duration-equalized vowels was clearly observed with respect to reducing the duration reliance on vowel perception. The duration effect was significant before training for both groups; however, the duration effect became non-significant after training only for the vowel training group. This result suggests that native Chinese listeners successfully reduced their over-reliance on vowel duration for English vowel perception with the training in which no duration cue was available. Previous studies also found that native Finnish and Greek listeners significantly improved their identification of duration-equalized vowel pair /I—i/ and speculated the perceptual weight shift from vowel duration to vowel formant spectrum [18, 19]. Results of the present study and the above previous studies could be further interpreted by Francis’s model on attention to dimension [20]. In this model, learning is treated as a pair of complementary attentional operations that serve to change the structure of the perceptual space to produce categorization. These operations were formalized in terms of a weight that stretches or shrinks the dimensions of perceptual contrasts; that is, perceptual training with equalized-duration vowels may change the structure of the perceptual space and shift the perceptual weight to other perceptual cues (e.g., spectral cues). In contrast, although general speech input had significantly improved native-Chinese listeners’ English vowel perception, it did not help reduce listeners’ dependence on duration for English vowel perception. For this group, the duration effect of vowel identification remained significant throughout the pre-training, post-training, and retention tests.

The equalized duration vowel training may trigger the compensation of the use of other perceptual cues (e.g., spectral cues) for non-native vowel perception. This study examined whether short-term vowel training with equalized duration vowels strengthened the association between vowel identification and vowel formant discrimination for non-native listeners. The results showed that for the training group the correlation between vowel identification and formant discrimination increased from non-significant to significant after vowel perception training, which was synchronized with the reduced duration effect from significant to non-significant. In contrast, the correlation between vowel identification and vowel formant discrimination did not change for the control group. Therefore, this study suggests that perception training with duration-equalized vowels may shift non-native listeners’ perceptual strategy from heavy reliance on duration cues to less heavy reliance, while placing greater reliance on spectral cues. However, after three months, such shifts in the perceptual strategies were not retained, indicating that short-term training may not be able to fundamentally and permanently change non-native listeners’ perceptual weights of acoustic cues for their English vowel perception.

## Limitations and future directions

First, only one individual vowel /ʌ/ was used in the present study for the vowel formant discrimination task primarily because of the concern with the length of the experiments. It usually took about 30 minutes to examine the formant discrimination of the F2 frequency of one vowel, and thus it will take a long time to include multiple vowels, especially for the pre- and post-training measures. However, to avoid potential bias, more vowels need to be included in the vowel formant discrimination task in future studies.

Second, multiple talkers were used to produce training stimuli; however, a previous study suggested that not all learners benefit from talker variability. Antoniou and Wong found that low-aptitude listeners performed worse in a tone perception task with multiple talkers when cognitive load was increased [34]. After comparing their study with the present one, we found that a secondary task was used to manipulate the cognitive load in Antoniou and Wong’s study, while the present study used only a single task. Moreover, a previous study showed that talker variability helped learning under the single-task condition [13]; thus, multiple talkers were chosen in the present study. To further clarify the role of the multiple-talker variability on non-native speech learning, more research is needed on the interaction of cognitive load and talker variability.

Third, the relatively short inter-stimulus interval (ISI) (400 ms in this study) may activate a ‘phonetic mode’ of processing in a vowel formant discrimination task for non-native listeners who might shift their preference for long ISI to short ISI after training [35]. More studies are needed to examine how ISI affects vowel formant discrimination, and how such an effect, if any, is modified by perceptual training for non-native listeners.

It also should be noted that the vowel formant discrimination of /ʌ/ significantly correlated with the performance of vowel identification in the equalized duration in the study by Mi et al. [9], but not in the pre-training test of this study. This discrepancy may be due to differences in the L2 experience in the two groups of participants. In the present study, participants started to learn English much later (11 or 12 years old), compared with the Mi et al. study in which some started as early as age six. We speculate that the relationship between formant discrimination and vowel identification may be affected by the English learning experience of listeners. Because we did not aim to replicate the Mi et al. study, the learning experience of listeners was not comparable between the two studies. More studies may be needed to examine the possible impact from L2 experiences on the correlation between vowel formant discrimination and vowel identification.

Lastly, results from this study show that vowel formant discrimination improved right after vowel identification training, but it did not last for a long time. Kewley-Port found that vowel formant discrimination could be improved right after vowel formant discrimination training [36]. These studies including the present one showed the instant effect of perceptual training on vowel formant discrimination; however, to our knowledge, no data has been documented on a retention effect of vowel formant discrimination training. In the future, longer training duration and more complex phonetic contexts may need to be included to enhance the retention effect. For example, a training protocol in vowel formant discrimination can be examined to determine whether increased sensitivity to a formant frequency change after training would improve vowel identification.

## Conclusions

Compared with increasing general English speech input, English vowel perceptual training with equalized duration not only improved native Chinese listeners’ English vowel identification and vowel formant discrimination, it also reduced their heavy reliance on vowel duration and improved their use of spectral cues in English vowel perception.

## Supporting Information

S1 Appendix Analyses and results of the logit transformed scores. (DOCX)
