# Supplementary material for: Shifting Perceptual Weights in L2 Vowel Identification after Training
Source: PLoS One. 2016 Sep 20;11(9):e0162876. doi: 10.1371/journal.pone.0162876 (PMC5029867; doi:10.1371/journal.pone.0162876)
Supplement: S1 Appendix — (DOCX) [file pone.0162876.s001.docx]

**Appendix：Analysis and Results of the Logit Transformed Scores**

**Improvement and retention in vowel identification**

**For trained vowels:** A three-way (within-subjects factors: vowel duration × test time; between-subjects factor: group) repeated-measures ANOVA was performed. The results showed significant main effects of test time (*F*_2, 52_ = 34.87, *p* < 0.001, η_p_^2^ = 0.573), vowel duration (*F*_1, 26_ = 17.73, *p* < 0.001, η_p_^2^ = 0.405), and significant interaction effects between the three factors (*F*_2, 52_ = 3.44, *p* = 0.040, η_p_^2^ = 0.117) and between test time and vowel duration (*F*_2, 52_ = 3.34, *p* = 0.042, η_p_^2^ = 0.115). However, no significant main effect of group (*p* = 0.327) and no significant interaction effects between test time and group or between vowel duration and group was found (*p* = 0.309; *p* = 0.290).

**For untrained vowels:** Similar ANOVA analysis was performed on untrained vowels. The results showed significant main effect of test time (*F*_2, 52_ = 12.47, *p* < 0.001, η_p_^2^ = 0.324), while no significant effects for vowel duration, group, and multi-factor interactions were observed (three order interaction: *p* = 0.429; interaction between duration and group: *p* = 0.693; interaction between time and group: *p* = 0.116; interaction between duration and time: *p* = 0.250).

**Training effect on vowel identification of a new talker**

**For trained vowels:** A two-factor (within-subjects factor: test time; between-subjects factor: group) ANOVA was conducted with the vowel identification score of equalized condition only as the dependent variable. The main effects of group and test time were significant (group: *F*_1, 26_ = 5.38, *p* = 0.029, η_p_^2^ = 0.171; test time: *F*_1, 26_ = 7.42, *p* = 0.011, η_p_^2^ = 0.222). The interaction effect between test time and group was not significant (*p* = 0.082).

**For untrained vowels:** Similar ANOVA analysis was performed on untrained vowels. No significant main effects were found (group: *p =* 0.073; test time: *p* = 0.712), and no significant interaction effect between test time and group was observed (*p* = 0.878).

**Duration effect change in vowel identification**

For the vowel training group, subsequent simple effect analysis of marginal significant interaction effects between the three factors in a three-way (within-subjects factors: vowel duration × test time; between-subjects factor: group) ANOVA revealed that there was a significant duration effect (more than 15%, *p* = 0.011) before the training, while there was no significant duration effect (less than -1%, *p* = 0.359) after the vowel training, indicating that the listeners in the vowel training group reduced the reliance on vowel duration for the identification task after training. In addition, non-significant duration effects (less than 2%, *p* = 0.549) in the retention test indicated that this reduced duration effect was maintained three months after training. In contrast, listeners in the control group consistently showed significant duration effects for the pre-training test (10%, *p* = 0.005), the post-training test (10%, *p* = 0.008), and the retention test (12%, *p* = 0.040), suggesting that a general English input such as video watching did not change Chinese listeners’ reliance on duration for vowel perception.
